# Salivary Amylase‐Responsive Buccal Tablets Wipe Out Chemotherapy‐Rooted Refractory Oral Mucositis

**DOI:** 10.1002/advs.202308439

**Published:** 2024-01-16

**Authors:** Yan Zhang, Taixia Wang, Xiulin Dong, Chunyan Zhu, Qiuxia Peng, Chang Liu, Yifeng Zhang, Fubo Chen, Kun Zhang

**Affiliations:** ^1^ Department of Medical Ultrasound and Department of Stomatology Shanghai Tenth People's Hospital Tongji University School of Medicine Tongji University NO. 301 Yan‐chang‐zhong Road Shanghai 200072 China; ^2^ Department of Pharmacy and Central Laboratory Sichuan Academy of Medical Sciences Sichuan Provincial People's Hospital University of Electronic Science and Technology of China No. 32, West Second Section, First Ring Road Chengdu 610072 China

**Keywords:** anti‐inflammatory, buccal tablets, refractory oral mucositis, salivary amylase response, wound healing

## Abstract

Oral mucositis (OM) is the most common and refractory complication of cancer chemotherapy and radiotherapy, severely affecting patients’ life quality, lowering treatment tolerance, and discouraging patient compliance. Current OM delivery systems mostly affect the comfort of patient use and lead to poor compliance and unsatisfactory effects. Herein, salivary amylases (SAs)‐responsive buccal tablets consisting of porous manganese‐substituted Prussian blue (PMPB) nanocubes (NCs), anti‐inflammatory apremilast (Apr) and starch controller have been engineered. PMPB NCs with large surface area can serve as carriers to load Apr, and their multienzyme‐mimicking activity enables them to scavenge reactive oxygen species (ROS), which thus synergize with Apr to mitigate inflammation. More significantly, the starch controller can respond to abundant SAs in the oral cavity and realize the cascade, continuous, and complete drug release after enzymatic decomposition, which not only aids with high tissue affinity to prolong the resistance time but also improves the comfort of use. The preclinical study reveals that contributed by the above actions, such buccal tablets mitigate inflammation, promote endothelium proliferation and migration, and accelerate wound healing for repressing chemotherapy‐originated intractable OM with positive oral microenvironment and shorter recovery time, thus holding high potentials in clinical translation.

## Graphical abstract

A composite buccal tablet consisting of porous manganese‐substituted Prussian blue nanocubes, anti‐inflammatory apremilast, and starch controller has been engineered, which enables the salivary amylases‐responsive release of active components to scavenge reactive oxygen species, mitigate inflammation, promote endothelium proliferation/migration, and accelerate wound healing for repressing chemotherapy‐originated intractable oral mucositis with the prolonged resistance time and improved comfort of use.

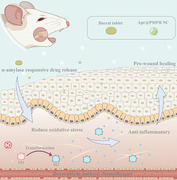

## 1 Introduction

It is widely accepted that cancer has severely endangered human health, and great efforts and all eyes have been devoted to cancer diagnosis and treatment. Nevertheless, the complications or syndromes after various treatments receive less attention compared to cancer diagnostics. Oral mucositis (OM) is one of the most severe complications of chemotherapy and head and neck radiotherapy.^[^ ^1^, ^2^, ^3^, ^4^ ^]^ Differing from other inflammation‐related diseases, chemotherapy or radiotherapy‐raised OM is refractory, thus resulting in the failure of a complete cure even with analgesia management. Patients with severe OM are more likely to suffer from oral microbial infection due to the immunocompromised state, some of which may escalate into systemic infection.^[^ ^5^, ^6^ ^]^ Consequently, the systemic infection decreases the therapeutic tolerance dose in tumor chemotherapy or radiotherapy, reversely reshapes the tumor microenvironment, and renders tumor resistance to chemotherapy or radiotherapy, which thereby lowers curative outcomes and survival rates.^[^ ^7^, ^8^ ^]^ Unfortunately, OM treatment remains a significant challenge in tumor treatment since there are still few efficient treatment methods available for patients to prevent or mitigate them.^[^ ^9^ ^]^ Although clinical data in a phase III multinational study showed that apremilast (Apr), an oral phosphodiesterase 4 inhibitor, could improve oral ulcers and multiple other outcomes of patients with leukoaraiosis syndrome,^[^ ^10^ ^]^ there are limited data on the efficacy and safety of Apr in the treatment of patients with refractory oral ulcers caused by radiotherapy or chemotherapy.

Generally, the pace of OM development depends closely on the toxicity of chemotherapy protocol. Given this, properly administered oral medications or biologic agents can boost patient compliance, especially for those patients with chronic diseases that require regular medication and long‐term observation.^[^ ^11^ ^]^ Therein, topical treatment is a reasonable choice for quick pain relief caused by OM.^[^ ^12^, ^13^ ^]^ Oxidative stress, inflammatory response, as well as wound healing, are identified as the potential mechanistic targets of OM in clinical practices, which can be well harnessed to engineer appropriate protocols or functional agents to repress OM progression.^[^ ^9^, ^14^, ^15^ ^]^ Nevertheless, influenced by saliva flushes and mouth movement, current commercial drugs are confronted with challenges including short residence time, fast degradation, and unsatisfactory wound healing.^[^ ^16^, ^17^ ^]^ Great attempts have been made to develop drug delivery platforms for oral applications, wherein those platforms with strong adhesion under wet conditions,^[^ ^18^ ^]^ prolonged drug release time, and facile drug penetration across the epithelial barriers are highlighted.^[^ ^19^ ^]^ Typically, inspired by the excellent underwater adhesion of marine lives, for example, mussels,^[^ ^18^ ^]^ barnacles, and tubeworms,^[^ ^20^ ^]^ researchers have developed several oral bioadhesives that partially satisfy the oral demands. Though these oral bioadhesives exhibit efficient properties in anti‐inflammatory and mucositis repair, these bioformulations encounter setbacks in clinical translation because of their poor comforts of use and compliance. The poor comport may impair patients' motivation to receive repeated treatments.^[^ ^21^ ^]^ On this account, ideal bioformulations should not only allow highly efficient OM removal but also take into consideration of use of comfort.

**Figure 1:**
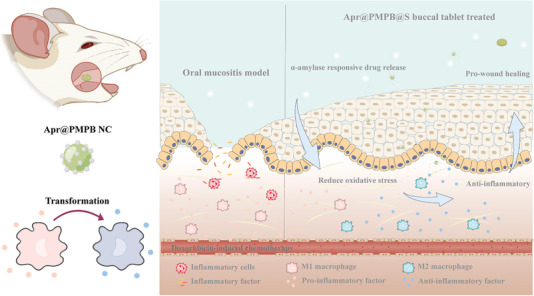
Schematic diagram of Apr@PMPB@S buccal tablet for anti‐inflammatory and wound‐healing on chemotherapy‐induced oral mucositis.

In this report, salivary amylases (SAs)‐responsive buccal tablets that utilized starch as a responsive unit to encompass porous manganese‐substituted Prussian blue (PMPB) nanocubes (NCs) have been engineered to uproot OM, as represented by inflammation elimination and wound heal (**Figure** **1**). Therein, PMPB NCs were leveraged to accommodate the anti‐inflammatory drug (i.e., Apr) to obtain Apr@PMPB NCs, and dextrin served as the binder to enable them to shape into the stable buccal tablet with starch controller (namely Apr@PMPB@S). Porous nanoparticles as carriers exhibit unparalleled advantages in the delivery and controlled release of drugs.^[^ ^14^, ^22^, ^23^ ^]^ They can be also engineered into functional agents capable of mitigating inflammation.^[^ ^24^, ^25^ ^]^ Inspired by it, PMPB NCs featured large surface area and high safety and possessed multienzyme activity including catalase, superoxide dismutase, and peroxidase.^[^ ^26^, ^27^, ^28^ ^]^ Moreover, they remarkably synergized with Apr to scavenge reactive oxygen species (ROS), mitigate inflammation, wipe out chemotherapy‐induced OM, and accelerate wound healing with a positive oral microenvironment and shorter recovery time (Figure 1). Among various controllable release means, the enzyme‐responsive strategy has emerged to be a popular one since it is biospecific, efficient, and biosafe.^[^ ^29^, ^30^ ^]^ In light of the facts that there are abundant SAs in the oral cavity and starch and its primary hydrolyzates are sensitive to α‐amylase, the continuous release of PMPB NCs and the cascade release of Apr were attained in response to SAs. These inspiring release properties were much preferable and applicable for oral applications of buccal tablets. Especially, the SAs‐responsive release manner and the high tissue affinity property of starch not only prolonged the resistance time of active components in the oral cavity compared to direct deglutition but also improved the comfort of use that bioadhesives failed to achieve. Moreover, all components including Prussian blue are biosafe and approved in clinics by the Food and Drug Administration (FDA). Collectively, such SAs‐responsive buccal tablets (Apr@PMPB@S) as a comprehensive oral drug delivery system integrated with excellent bioavailability with highly efficient therapeutic efficacy for OM elimination. This pre‐clinical study indicates the high potential of such SAs‐responsive buccal tablets in removing the refractory OM after chemotherapy or radiotherapy.

## 2 Results

### 2.1 Synthesis of Biomimetic Apr@PMPB NCs and Apr@PMPB@S Buccal Tablets

**Figure 2:**
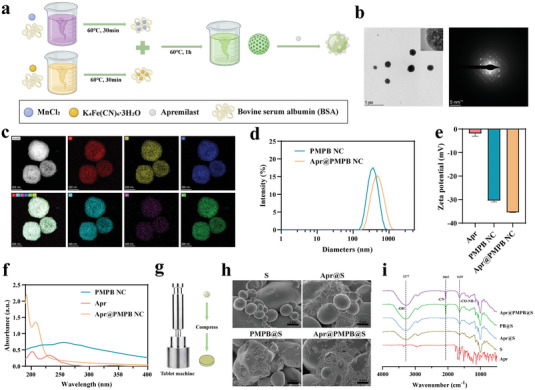
Synthesis procedure and characterization of the biomimetic PMPB NC and Apr@PMPB NC. a) Synthetic procedure of PMPB NCs and Apr@PMPB NCs. b) TEM images and electron diffraction pattern of PMPB NCs. c) The element mapping images of PMPB NCs, including C, Fe, K, Mn, N, and S within the selected region of interest. d) Hydrodynamic diameter distribution curves of PMPB NCs and Apr@PMPB NCs. e) Zeta potentials of PMPB NCs and Apr@PMPB NCs. f) UV–vis spectra of free Apr, PMPB NCs, Apr@PMPB NCs. Data are expressed as mean ± SD (*n* = 3). g) Construction procedures of the Apr@PMPB@S buccal tablets. h) SEM images of S, Apr@S, PMPB@S, and Apr@PMPB@S. i) FTIR spectra of S, Apr@S, PMPB@S, and Apr@PMPB@S.

PMPB NCs were synthesized through biomineraization according to the previous study (**Figure** **2**a).^[^ ^31^ ^]^ In this method, bovine serum albumin (BSA) performed as the stabilizer as well as structure‐directing agent to bind with Mn^2+^ and [Fe(CN)_6_]^4−^ ions, followed by Mn&Fe co‐precipitation to yield PMPB NCs. Transmission electron microscopy (TEM) images show that the synthesized PMPB NCs exhibit a well‐defined morphology with an average diameter at around 200 nm and a high degree of crystallinity (Figure 2b). Typical atom mapping images indicate the distribution positions of K, Fe, S, Mn, N, etc. (Figure 2c). X‐ray photoelectron spectra (XPS) analysis (Figure S1, Supporting Information) verifies the valences of Mn and Fe in PMPB NCs. Both TEM and XPS indicate the successful synthesis of PMPB NCs, which is consistent with the previous report.^[^ ^31^ ^]^ Besides scavenging ROS, PMPB NCs also serve as carriers to load and deliver Apr because PMPB NCs are imparted with large surface area and appropriate pore diameter (Figure S2, Supporting Information). Herein, both PMPB NCs and Apr were mixed and stirred overnight to obtain Apr@PMPB NCs, and the morphology and size failed to vary (Figure S3, Supporting Information). The loading of Apr results in the increase of particle size (Figure 2d and Figure S4, Supporting Information), and also brings about the change of zeta potential from −30.46 to −35.46 mV (Figure 2e), which forcefully confirms the successful entrapment of Apr in PMPB NCs. UV–vis spectra also verify the successful loading of Apr in PMPB NCs (Figure 2f), and the loading amount of Apr in the final Apr@PMPB NCs is calculated to be 18.41%. As for the preparation of buccal tablets, binders (e.g., dextrin) and response units (e.g., starch) were added and mixed with Apr@PMPB NCs, followed by routine compression on the tablet machine to construct the Apr@PMPB@S buccal tablets. As for the preparation of buccal tablets, binders (e.g., dextrin) and response units (e.g., starch) were added and mixed with Apr@PMPB NC, followed by the routine compression on the tablet machine to obtain the Apr@PMPB@S buccal tablets (Figure 2g). It is clearly found that PMPB NCs and Apr@PMPB NC are confined and adhered to starch in the tablets (Figure 2h), and the successful entrapment of Apr is further verified by an emerging and representative peak corresponding to ‐CN at 2065 cm^−1^ (Figure 2i).

### 2.2 Extracellular ROS Scavenging Test

ROS plays a series of roles in either physiological or pathological conditions to regulate biological activities. It is well known that excessive ROS levels may induce severe oxidative damage and result in inflammatory reactions that finally lead to some chronic diseases.^[^ ^32^, ^33^ ^]^ As a result, ROS scavenging is of great significance in anti‐inflammation and anti‐oxidative therapy.^[^ ^34^, ^35^, ^36^, ^37^ ^]^ In recent years, with the rapid development of nanotechnology, various nanomaterials with the extraordinary capability of ROS scavenging have been designed to repress inflammatory progression. Herein, we used UV–vis spectrophotometry to evaluate the extracellular ROS scavenging ability of PMPB NCs.

**Figure 3:**
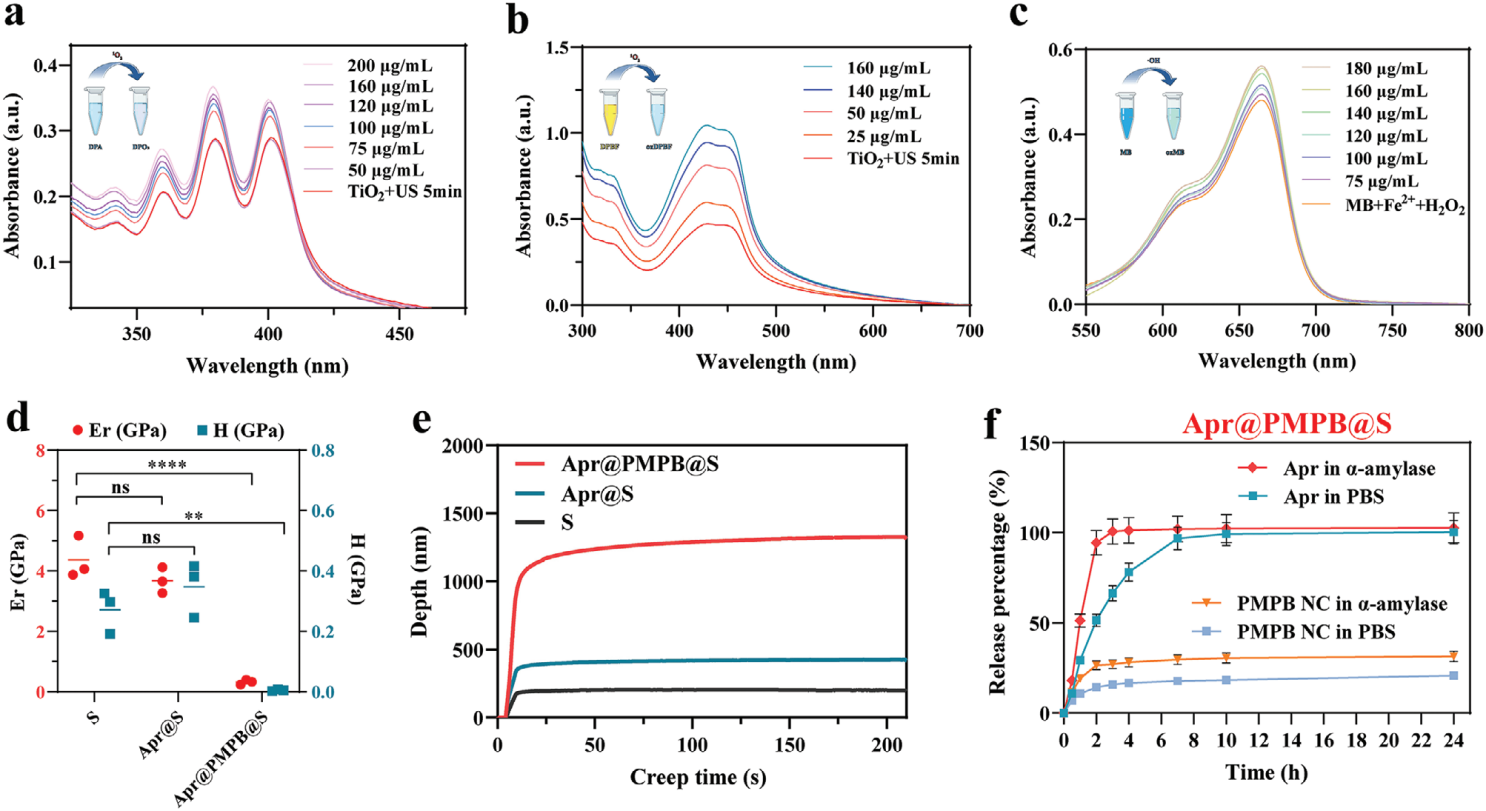
Extracellular ROS‐scavenging ability and α‐amylase responsive drug release analysis. a) Schematic on how ^1^O_2_ can degrade DPA and DPA can reflect ^1^O_2_ level, and the corresponding UV–vis absorbance spectra of DPA degradation triggered by sonocatalysis of TiO_2_ in the presence of PMPB NCs with varied concentrations. b) Schematic on how ^1^O_2_ can degrade DPBF can reflect ^1^O_2_ level, and the corresponding UV–vis absorbance spectra of DPBF degradation triggered by sonocatalysis of TiO_2_ in the presence of PMPB NCs with varied concentrations. c) Schematic on how ·OH can degrade MB and MB can reflect ·OH, and the corresponding UV–vis absorbance spectra of MB degradation triggered by Fenton reaction induced by Fe^2+^ and H_2_O_2_ in the presence of PMPB NCs with varied concentrations. d) Hardness (H) and elastic moduli (Er) of S, Apr@S and Apr@PMPB@S. e) Creep curves of S, Apr@S, and Apr@PMPB@S. f) Time‐dependent release profiles of Apr and PMPB NC from Apr@PMPB@S buccal tablets with and without adding α‐amylase. Data are expressed as mean ± SD (*n* = 3). One‐way analysis of variance (ANOVA) was used for comparisons among multiple groups (“ns”‐no significance, *p < 0.05, **p < 0.01, ***p < 0.001, ****p < 0.0001).

To assess the ROS scavenging, TiO_2_ nanoparticles that feature extremely high chemical stability were used as the most commonly used sonosensitizer to produce ROS via the sonocatalysis process.^[^ ^38^ ^]^ Moreover, 9,10‐diphenylanthracene (DPA) and 3‐diphenylisobenzofuran (DPBF) were used as probes to detect ^1^O_2_ levels in the course of TiO_2_‐mediated sonocatalysis in the absence or presence of PMPB NCs. DPA and DPBF can propel endoperoxide formation once they react with ^1^O_2_, leading to a remarkable decrease in the relative absorbance intensities of DPA and DPBF. UV–vis spectra show that the absorbance of DPA at 355 nm gradually ascends as the concentration of PMPB NCs increases (**Figure** **3**a). Similarly, the absorbance of DPBF at 455 nm rises in response to the concentration of PMPB NCs (Figure 3b), indicating the robust ^1^O_2_ scavenging ability of PMPB NCs.

Furthermore, another ROS was monitored using methylene blue (MB) since MB has been recognized as the probe of ·OH. Herein, the Fenton reaction between H_2_O_2_ (1 mM) and Fe^2+^ (100 µg•mL^−1^) was used to produce ·OH. It is clearly found that the absorbance intensity of MB at its characteristic peak declines, and the introduction of PMPB NCs rescues MB degradation by ·OH (Figure 3c). The higher the PMPB NC concentration is, the stronger the rescue ability is. All these results unveil the excellent scavenging ability of PMPB NCs against ROS, which is expected to benefit anti‐inflammation and OM recession.

### 2.3 Mechanical Property Assessment

Mechanical properties of the buccal tablets with different components were inspected using the nanoindentation method, wherein the correlation between indenter displacement (*h*) and load pressure (*P*), elastic modulus (*Er*) / hardness (*H*), and creep curves versus holding time can be recorded or obtained, respectively. During the loading‐unloading process, the load–displacement (P–h) curves of the S, Apr@S, and Apr@PMPB@S are provided in Figure S5, Supporting Information, wherein Apr@PMPB@S exhibits the largest deformation. Furthermore, Er and H values of various samples are obtained from the analysis of the obtained *P–h* curves. In detail, compared with S and PMPB@S, Apr@PMPB@S buccal tablets display a significantly decreased H (**p = 0.0047) and Er (****p < 0.0001) (Figure 3d), indicating the brittleness decline and the local viscoelasticity rise, which are much preferable for inducing the recovery of broken oral mucosa.^[^ ^39^ ^]^ Typical curves of creep depth versus holding time at selected penetration depths during nanoindentation show that all curves exhibit two distinct phases, that is, stages I and II (Figure 3e).^[^ ^40^ ^]^ Compared with S and Apr@S, the creep displacements of the Apr@PMPB@S increase. This phenomenon is attributed to that the binding and adhesion of PMPB NC on starch exert specific influences on the generation and movement of shear bands during the creep process and thus promote local plastic deformation.

### 2.4 Salivary Amylase‐Responsive Drug Release Test

Before the in vivo therapeutic assay, we first investigated the adhesive properties and the SAs‐responsive drug release behaviors of such buccal tablets. As shown in Figure S6, Supporting Information, the as‐prepared Apr@PMPB@S buccal tablets that undergo mixing and compression can stably attach to different surfaces ranging from plastics, paper fibers, and biological tissue surfaces, which will prolong the resistance time of buccal tablets in the oral cavity and avoid the rapid deglutition before the complete release of active components. The obtained Apr@PMPB@S tablets remain stable in PBS but progressively disintegrate when they incubate with α‐amylase (Figure S7a, Supporting Information), and longer incubation time proceeded, more complete decomposition is reached (Figure S7b, Supporting Information). Subsequently, we investigated the in vitro release profiles of Apr and PMPB NCs from Apr@PMPB@S buccal tablets. Results show that the environment containing α‐amylase decomposes starch and destroys buccal tablets to accelerate and allow more PMPB NCs release from the buccal tablets, and concurrently trigger the continuous complete release of Apr (Figure 3f). In contrast, without PMPB NC entrapment, Apr@S tablets rapidly respond to α‐amylase and bring about the burst Apr release form Apr@S (80% release within 10 min) (Figure S8, Supporting Information), disabling Apr to continuously exert the therapeutic effects on oral mucositis. These results successfully validate the SAs‐responsive release properties of active components from buccal tablets, which also denotes that the complete Apr release and more PMPB NCs release within a shorter time is much more beneficial for OM treatment in case of rapid deglutition.

### 2.5 In Vitro Cytotoxicity, Intracellular Pro‐Migratory and Anti‐Oxidation Explorations

**Figure 4:**
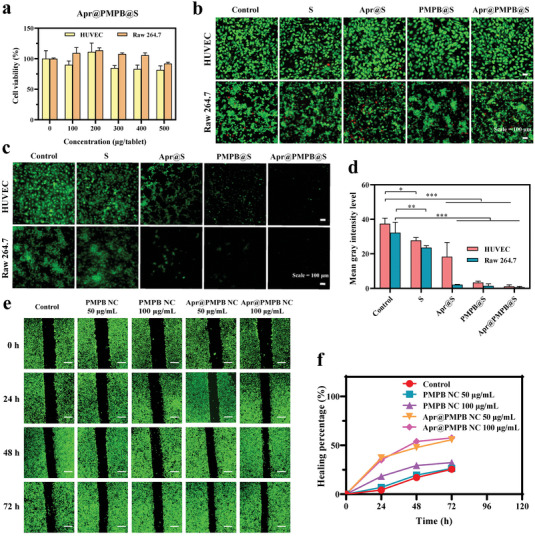
Evaluations on cytotoxicity, cell migration, and oxidative stress alleviation. a) Cell viability of HUVECs and Raw 264.7 treated with Apr@PMPB@S tablets. b) CLSM images of HUVECs and Raw 264.7 cells stained with Live/Dead after treatments with S, Apr@S, PMPB@S, and Apr@PMPB@S tablets, scale bar: 100 µm. c) CLSM images and d) quantitative data of Raw 264.7 cells and HUVECs stained with the ROS indicator, that is, DCFH‐DA probe after different treatment, and scale bar: 100 µm. e) Fluorescence images and f) quantitative scratch closure rates of Calcein‐AM‐stained HUVECs in the scratch assay for assessing migration images, and scale bar: 500 µm. Data are expressed as mean ± SD (*n* = 3). ANOVA was used for comparisons among multiple groups (*p < 0.05, **p < 0.01, ***p < 0.001).

Prior to assessing the safety of Apr@PMPB@S buccal tablets (i.e., Apr@PMPB@S), their active components (PMPB and Apr@PMPB) were assessed in in vitro tests. It has been widely accepted that good biocompatibility and non‐cytotoxicity are the prerequisites for deciding the appropriateness of some delivery carriers in biological applications. We evaluated the in vitro cytotoxicities of PMPB and Apr@PMPB NCs by CCK‐8 assay on both HUVECs and Raw 264.7 cells to figure out the safe range of PMPB NCs for use. After co‐culturing with PMPB or Apr@ PMPB NCs for 24 h, the survival rates of HUVECs and Raw 264.7 cells are above 80% within 100 µg•mL^−1^ (all *p*‐values <0.05) (Figure S9, Supporting Information). Therefore, the desired concentration scopes of PMPB NCs or Apr@PMPB NC that can be used for the following treatments are narrowed to the window (≈50 to 100 µg•mL^−1^). Further live‐dead cell experiments verify the safe range (Figure S10, Supporting Information), and results confirm that PMPB NCs and Apr@PMPB NCs have good cytocompatibility within 100 µg•mL^−1^ for biological applications (e.g., tissue regeneration). Subsequently, the in vitro cytotoxicity of Apr@PMPB@S buccal tablets was assessed to figure out the safe range for guiding in vivo experimental treatment. Buccal tablets were placed in the transwell cell culture room and then co‐cultivated with cells. The survival rates of both HUVECs and Raw 264.7 are all above 80% even though the dose of Apr@PMPB NC in the Apr@PMPB@S tablets reaches 500 µg (**Figure** **4**a). Live‐dead cell experiments also verify the high cytocompatibility of Apr@PMPB@S within 500 µg Apr@PMPB NC (Figure 4b).

Subsequently, intracellular ROS scavenging was examined so as to avoid oxidative stress damages that closely correlate with wound healing. Herein, 2′,7′‐dichlorofluorescin diacetate (DCFH‐DA) was utilized to trace the intracellular ROS variation in Raw 264.7 cells. Lipopolysaccharide (LPS) stimulates Raw 264.7 cells to give birth to higher intracellular ROS levels than no‐treated cells (control), which is designated as the model group (Figure S11a, Supporting Information). It is found that the introduction of PMPB NCs into Raw 264.7 cells in the model group considerably decreases the intracellular ROS level, indicating that the PMPB NCs can scavenge intracellular ROS. The ROS scavenging ability is in line with the concentration of PMPB NCs. More significantly, Apr@PMPB NCs at 100 µg•mL^−1^ bring about the largest decline amplitude of ROS level, revealing that Apr entrapment synergistically potentiates the anti‐oxidation effect of PMPB NCs. The corresponding quantitative data show identical results (Figure S11b, Supporting Information). Intriguingly, even though the Apr@PMPB active components are encapsulated in starch, their enhanced ROS scavenging ability is inherited, imparting Apr@PMPB@S buccal tablets with the strong ability to scavenge ROS. Results show that the ROS levels in both Raw 264.7 cells and HUVEC are the lowest upon incubation with Apr@PMPB@S buccal tablets (all *p*‐values <0.05) (Figure 4c,d).

The above intracellular anti‐oxidation property and pro‐migratory ability of Apr@PMPB NCs will take the dominant responsibility for wound healing and benefiting OM treatment. As expected, directional migration of HUVECs to wounds is highly desirable since these HUVECs can induce angiogenesis at the injured sites and thus accelerate the healing process. As is shown in Figure 4e, more cell migrations occur in PMPB NCs‐treated groups in comparison to the control group. In particular, Apr@PMPB NCs‐treated groups exhibit the greatest pro‐migratory property, whereas the Apr@PMPB NCs‐treated groups receive the narrowest scratch (or wound). The quantitative analysis further indicates more wound closure areas in the Apr@PMPB NCs‐treated groups than either the control group or PMPB NCs‐treated groups (Figure 4f).

### 2.6 In Vivo Therapeutic Efficacy and Biosafety Analyses of Different Tablets

**Figure 5:**
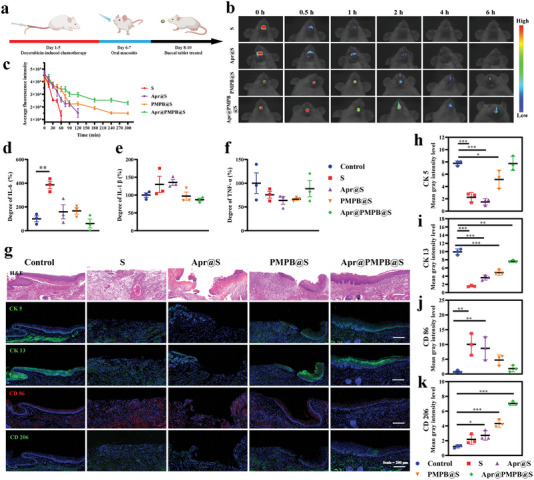
In vivo therapeutic evaluation of OM using Apr@PMPB@S buccal tablets to evaluate wound healing on chemotherapy‐induced OM model. a) Schematic diagram for depicting the construction and treatment strategies of chemotherapy‐induced OM wound models. b) In vivo fluorescence images of Cy5.5‐labeled Apr@PMPB@S buccal tablets and control buccal tablets (including S, Apr@S, and PMPB@S tablets) under PBS infiltration. c) Corresponding fluorescence intensity of Apr@PMPB@S buccal tablets and control buccal tablets. d) Relevant levels of IL‐6, e) IL‐1β, and f) TNF‐α in the serum of the Kunming mice after different treatments. g) H&E staining and immunohistochemical staining images of regenerated oral ulcers stained with anti‐cytokeratin 5 rabbit monoclonal antibody (CK 5, green), anti‐cytokeratin 13 rabbit polyclonal antibody (CK 13, green), and CD 86 polyclonal antibody (CD 86, red) and CD 206 polyclonal antibody (CD 206, green) at day 10. Nuclei (blue) were stained with DAPI. scale bar: 100 µm. h–k) Mean gray intensity levels of h) CK 5, i) CK 13, j) CD 86, and k) CD 206 immunohistochemical staining images in Kunming mice after different treatments. Data are expressed as mean ± SD (*n* = 3). ANOVA was used for comparisons among multiple groups (*p < 0.05, **p < 0.01, ***p < 0.001).

To evaluate the in vivo therapeutic efficacy of Apr@PMPB@S buccal tablets against OM, we established an OM model on mice induced by cancer chemotherapy. After the OM model establishment (Figure S12, Supporting Information), model mice were randomly divided into four experimental groups. Additionally, a group of healthy mice was set as a control group to inspect the therapeutic efficacy of different tablets by comparing experimental groups with the control group. Apr@PMPB@S buccal tablets (500 µg nanoparticles) were administered to OM mice under isoflurane anesthesia once a day within successive three days (**Figure** **5**a). Contributed to the long residence time, the obtained tablets are resistant to rapid degradation and flush away, enabling the continuous release of active units including PMPB NCs and Apr in comparison to other groups (Figure 5b,c). Intriguingly, three proinflammatory cytokines (e.g., IL‐1β, IL‐6, and TNF‐α) in the serum of the Kumming mice after different treatments were detected (Figure 5d–f). The pro‐inflammatory cytokines (e.g., IL‐6 and IL‐1β) are significantly down‐regulated in the Apr@PMPB@S‐treated group (**p = 0.0025) (Figure 5d,e), uncovering that Apr@PMPB@S buccal tablets can effectively attenuate inflammation in plaques. Additionally, the treatment compliance was monitored, and treated mice exhibited no rebellion behaviors such as nervous tension, tablet chewing, or tablet spitting during the treatment process, suggesting excellent treatment compliance and oral comfort.

H&E staining and immunofluorescence staining of CK 5 and CK 13 were performed to evaluate the inflammation and epithelium recovery, wherein CK 5 targeted the surface layer and CK 13 stained the intermediate layer, respectively.^[^ ^18^, ^20^, ^41^ ^]^ The broken ulcer with a disordered orientation in the epithelium verifies the successful construction of the OM model (Figure 5g). The wounded areas treated with PMPB@S or Apr@S buccal tablet group are partially repaired, exhibiting obvious wound closure and epithelium regeneration. Remarkably, the layers treated by the Apr@PMPB@S buccal tablets display almost complete epithelium coverage that approaches the normal group. Furthermore, immunofluorescence staining for CK 5 and CK 13 also manifested better epithelium coverage at the site of OM in the PMPB@S‐treated group and almost complete coverage in the Apr@PMPB@S‐treated group (all *p‐*values <0.05) (Figure 5g–i), but fail to be observed in Apr@S. This phenomenon is partially attributed to the burst release of Apr in response to SAs’ failure to continuously work on oral mucositis.

In recent reports, overexpressed ROS scavenging can reduce inflammation and accelerate the wound healing process.^[^ ^42^, ^43^, ^44^, ^45^ ^]^ To explore whether the Apr@PMPB@S buccal tablets can regulate inflammation in vivo, immunofluorescence staining of CD 86 and CD 206 was utilized to monitor the macrophage polarization because CD 86 and CD 206 are the markers of M1‐type and M2‐type macrophages, respectively. Compared with the OM model group, a drastic reduction in the percentage of M1‐type macrophages is observed in the Apr@PMPB@S treated group (all *p‐*values <0.01) (Figure 5g,j), and coincidently Apr@PMPB@S treatment promotes the polarization of M2‐type macrophages (all *p‐*values <0.05) (Figure 5g,k). The above results reveal that the Apr@PMPB@S buccal tablets can elevate the polarization of M2‐type macrophages and promote OM regeneration, eventually facilitating wound healing.

### 2.7 Biosafety Evaluation of Different Patches (Configurations) In Vivo

The injection of DOX in the model group and the tablets‐treated groups resulted in a decrease in body weight compared with the healthy group, showing the severe side effects of chemotherapy (Figure S13, Supporting Information). As for in vivo histocompatibility and biosafety evaluation of different tablets, major organs (the heart, liver, spleen, lung, and kidney) were sliced for H&E staining. Neither obvious tissue damage nor pathological changes in these major organs during the therapeutic process of S, Apr@S, PMPB@S, and Apr@PMPB@S tablets are observed (Figure S14, Supporting Information). Finally, blood analysis demonstrates the neglectable changes in routine blood and biochemistry indexes after treatments with S, Apr@S, PMPB@S, and Apr@PMPB@S tablets (Figure S15, Supporting Information). All these results definitely indicate the high biosafety and biocompatibility of Apr@PMPB@S tablets, paving a solid foundation for clinical translation.

## 3 Discussion

Cancer therapy has increased the most attention, but the side effects or complications received less attention. OM is one of the most common side effects of chemotherapy and radiotherapy. Patients with severe OM are more likely to suffer from oral microbial infection due to the immunocompromised state, some of which may escalate into systemic infection that may lower curative outcomes and survival rates.

Refractory OM continues to set obstacles for patients who are undergoing cancer treatment, for example, lowering therapeutic tolerance dose and patient compliance.^[^ ^6^, ^9^ ^]^ Emerging advances in oral drug delivery platforms are improving the overall performance of oral drugs, allowing the advent of more sophisticated designs. Biomaterials‐based multi‐component delivery systems with multi‐functionalization remain an important obstacle in the design of biomedical applications.^[^ ^11^ ^]^ Compounds capable of impairing OM in clinical development have potential targets including oxidative stress relief and anti‐inflammatory response reinforcement, which, nevertheless, are still confronted with unsatisfactory healing outcomes.^[^ ^14^, ^15^, ^16^, ^17^ ^]^ To this end, we utilized the ROS scavenging capability of PMPB NC to unit with the anti‐inflammatory effect of Apr, achieving enhanced anti‐inflammatory and wound‐repairing effects against OM. PMPB NCs armed with rich multienzyme activities can eliminate ROS in the lesion area to proceed to mitigate inflammation, and Apr itself functions as an anti‐inflammation drug to repress inflammation progression. Thereby, the two active components synergized to relieve inflammation symptoms of refractory OM and favor OM treatment. Notably, the eventual formulation (Apr@PMPB@S) outperforms Apr@S in treating OM. These results explain that the introduction of PMPB indeed not only loads Apr and boosts the viscoelasticity to enable the continuous release rather than burst release, but also aids Apr in scavenging ROS and mitigating inflammation.

Very recently, several newly developed nanoparticle‐based buccal delivery systems, for example, mussel‐inspired mucoadhesive film,^[^ ^18^ ^]^ and barnacle‐inspired Janus patch,^[^ ^20^ ^]^ have been obtained to achieve strong adhesion to wet conditions in case of deglutition before completely pesticide effect. However, they might exert negative impacts on the comfort of use and compliance, further hampering clinical translation. Despite the significant breakthrough in targeting specific microbial and biofilm for treating buccal diseases,^[^ ^46^ ^]^ these engineered nanoparticles exerted negative impacts on the comfort of use and compliance. Appealingly, the SAs‐responsive buccal tablets were engineered to handle them, and they could realize the continuous and complete release of active PMPB NCs and Apr in case of immoderate time‐induced discomfortableness. Therefore, such an α‐amylase‐responsive drug release ability ensured a complete treatment effect with high biosafety as well as comfort of use. More significantly, the basic design strategy can serve as a general method to facilitate the clinical translation of drug delivery platforms for oral and gastrointestinal applications. It should be emphasized that the clinical transformation of Apr@PMPB@S demands consideration and optimization of more factors, for example, the preparation procedures including synthetic complexity, easy‐to‐handle property, loading ratio, delivery or accumulation efficiency in the lesion, and so on.

## 4 Conclusion

In summary, we successfully constructed SAs‐responsive buccal tablets as an oral mucosal drug delivery system to treat OM. The ROS scavenging ability of one component (i.e., PMPB NCs) in such buccal tablets synergized with another active component (i.e., Apr) to contribute to the removal of oxidative stress and inflammation mitigation. The Apr‐loaded buccal tablets accelerated wound‐healing of chemotherapy‐induced OM by the continuous and complete release of Apr in response to α‐amylase. They not only prolonged the resistance time in the oral cavity compared to direct deglutition but also improved the comfort of use compared to bioadhesives. Moreover, the continuous and complete release of Apr and PMPB NCs determined that Apr@PMPB@S buccal tablets favored inflammation inhibition and OM elimination with enhanced wound healing. This therapeutic mechanism was validated to correlate with the expedited macrophage polarization toward M2 phenotype and the reduced proinflammatory factor secretions. All FDA‐approved components guarantee biosafety, thus dictating that this biocompatible Apr@PMPB@S tablet can be considered as a candidate for clinical translation as an OM drug.

## 5 Experimental Section

### Materials

K_4_[Fe(CN)_6_], MnCl_2_, 2′,7′‐dichlorofluorescin diacetate (DCFH‐DA), and lipopolysaccharide (LPS) were purchased from Sigma‐Aldrich (USA). Apr was purchased from Shanghai Aladdin Biochemical Technology Co., Ltd. Penicillin‐streptomycin solution, trypsin‐EDTA solution, fetal bovine serum (FBS), and Dulbecco's modified Eagle's medium (DMEM) were purchased from Gibco (USA). Antifade mounting medium with DAPI, dimethyl sulfoxide (DMSO), and BSA were purchased from Beyotime (China). Cell Counting Kit‐8 and Calcein‐AM/PI double staining kit were purchased from Dojindo (Japan). Laser confocal scanning microscopy (CLSM)‐specific dishes (35 × 10 mm) were purchased from Corning Inc (New York). All other chemical reagents were analytical reagents. Deionized (DI) water was used throughout the research.

### Synthesis of PMPB NC

K_4_[Fe(CN)_6_] (6.25 mg) and BSA (50 mg) were dispersed in 10 mL of distilled water under magnetic stirring for 0.5 h at 60 °C, denoting as solution A. MnCl_2_ (16.88 mg) and BSA (50 mg) were dispersed in 10 mL deionized water distilled water under magnetic stirring for 0.5 h at 60 °C (denoted as solution B). Then, solution A was added slowly into solution B and stirred for another 1 h at 60 °C. The mixed solution was cooled and aged at room temperature for 24 h. The products were collected by centrifugation (10 000 rpm, 10 min) and washed with distilled water several times. Ultimately, PMPB NCs were acquired and stored at 4 °C for later use.

### Construction of Apr@PMPB NC and Apr@PMPB@S Buccal Tablets

10 mg PMPB NC was dispersed in DMSO, and then Apr (5 mg) was slowly added and stirred at room temperature for 24 h. Then, the products were centrifuged and washed with distilled water several times. The Apr@PMPB NCs were stored at 4 °C for the following use.

The drug loading ratios of Apr were calculated by Equation (1).^[^ ^25^, ^47^ ^]^

(1)

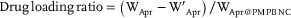




where *W*_Apr_ denotes the total mass of added Apr; *W*’_Apr_ indicates the mass of Apr in the supernatant; *W*_Apr@PMPB NC_ is the mass of Apr@PMPB NC.

Apr@PMPB (500 µg) in 10 µL of ultra‐pure water was firstly mixed with 3 mg dextrin and starch (10 mg), and then placed in the tablet machine compressed to construct the Apr@PMPB buccal tablets (Apr@PMPB@S). The same procedure was applied to yield the PMPB buccal tablets (PMPB@S) without adding Apr, and equivalent Apr was added to dextrin and starch to yield Apr@S buccal tablets without adding PMPB NCs.

### Characterization of the Apr@PMPB NC

The formation of PMPB NC was measured by TEM (FEI Talos F200X transmission electron microscope) and scanning electron microscopy (SEM) micrographs. The corresponding element mapping was intuitively demonstrated on a field‐emission Magellan 400 microscopy. Zeta potential and particle size distribution were measured on the Zetasizer Nano series (Nano ZS90) to detect the changes from PMPB NCs to Apr@PPB NCs. UV–vis absorbance spectra were recorded using a UV‐3600 Shimadzu spectrometer. To further analyze the chemical status of the samples, XPS analysis was performed on Thermo ESCALAB 250Xi (Thermo Scientific, US). N_2_ adsorption and desorption isotherms and pore diameter distribution were obtained on ASAP 2460 (Micromeritics Instrument Corp., USA).

### Nanoindentation Tests

Nanoindentation tests were carried out to obtain the *P–h* curves, Er/H, and time‐dependent creep curves of various samples to evaluate their mechanical properties. In detail, a rigid indenter (the standard Berkvoich indenter) with a regular shape was pressed into the surface of the sample under a gradually increased external force, and then the force was gradually unloaded after the external force (or displacement) reached a predetermined peak. During the loading‐unloading process, the displacement of the indenter and the load pressure applied by the indenter were recorded with the help of a high‐precision load‐displacement testing technique, and concurrently, the creep depth versus holding time was recorded. The elastic modulus (Er) and hardness (H) of samples were obtained from the analysis of the *P–h* curve.

### ROS Scavenging Activity Evaluation

^1^O_2_ and ·OH were selected as representative ROS to investigate the ROS scavenging activities of PMPB NC. Briefly, 9,10‐diphenylanthracene (DPA) (100 µg•mL^−1^) was added into the ^1^O_2_‐containing solution, which was produced by the inorganic sonosensitizer titanium dioxide (TiO_2_) (200 µg•mL^−1^) under ultrasonic irradiation. Meanwhile, the ^1^O_2_ solution was incubated with the PMPB NC at a series of concentrations for 5 min. The supernatant (1 mL) was collected to record absorption at 335 nm hence to evaluate the ^1^O_2_ scavenging effect as a function of PMPB concentration. 3‐diphenylisobenzofuran (DPBF) was another indicator for the detection of ^1^O_2._

Methylene blue (MB) (250 µg•mL^−1^) was used as the indicator to assess ·OH scavenging ability of PMPB NCs. Fenton reaction between H_2_O_2_ (1 mM) and Fe^2+^ (100 µg•mL^−1^) produced the ·OH‐containing solution, during which PMPB NCs with varied concentrations were added. The supernatant (1 mL) was collected to record absorbance intensity at 666 nm.^[^ ^25^ ^]^

### Release of Apr and PMPB NCs from Apr@PMPB@S Buccal Tablets

The release of Apr and PMPB NC in Apr@PMPB NC buccal tablet was detected by a dialysis method at PBS with and without α‐amylase at 37 °C. In brief, 15 buccal tablets (i.e., Apr@PMPB@S) were placed into a dialysis bag (MWCO  =  3500 Da) filled with 6 mL of α‐amylase‐included or α‐amylase‐free PBS solution. Afterward, the dialysis bag was immersed in 30 mL of PBS and stirred under 100 rpm at 37 °C. At certain time intervals, the absorbance intensity of UV–vis spectra of Apr at 230 nm was detected to determine the release profiles of Apr.

As for PMPB release, no dialysis bag was used, and direct collection at certain time intervals was carried out for ICP‐AES analysis, and afterward, the release profiles of PMPB were determined according to the Mn levels

### In Vitro Study

Cell Counting Kit‐8 (CCK‐8) and Live/Dead assays were performed to evaluate the effect of PMPB NCs and Apr@PMPB NCs on cytocompatibility. Human umbilical vein endothelial cells (HUVECs) and mouse macrophage Raw 264.7 cells were pre‐seeded in 96‐well plates at a density of 1 × 10^4^ cells per well, respectively, and cultured under 5% CO_2_ environment at 37 °C overnight. Both cell lines were further incubated with fresh culture medium containing PMPB NCs at different concentrations for 24 and 48 h, respectively. Subsequently, the excessive medium was removed, and cells were incubated with Cell Counting Kit‐8 (100 µL, V_CCK‐8_:V_DMEM_ = 1:9) medium for 60 min and then measured on a microplate reader at the wavelength of 450 nm to calculate the cell viabilities. The cell viability was calculated according to Equation (2).^[^ ^42^, ^48^ ^]^

(2) $$\mathrm{Cell}\;\mathrm{viability}\;\left(\%\right)=\left[\left({\mathrm{Ab}}_{\mathrm{sample}}-{\mathrm{Ab}}_{\mathrm{blank}}\right)/\left({\mathrm{Ab}}_{\mathrm{control}}-{\mathrm{Ab}}_{\mathrm{blank}}\right)\right]\times 100\%$$

Then, a Live/Dead cell assay was conducted using a 24‐well plate at a density of 1.5 × 10^4^ cells per well. Cells were adhered to the plate for 24 h, then 500 µL of fresh culture medium containing PMPB NCs or Apr@PMPB NCs was added and incubated for another 24 h. After staining with calcein‐AM/propidium iodide (PI) dyes for 15 min, the fluorescent images were captured under a fluorescence microscope (Nikon DS‐Riz; Japan) at different wells.

A scratch assay was performed to evaluate the migration of HUVECs. In brief, HUVECs were cultured in 6‐well plates with DMEM containing 10% FBS until 100% confluency was realized. Then, a pipette tip was used to create a linear wound, after which the cell debris was washed with PBS solution three times. The scratched cells were then cultured with fresh FBS‐free DMEM with different contents for different time periods. Phase contrast images were obtained by an inverted fluorescent microscope. The wound size was measured by Image J software, and the wound healing percentage was calculated using Equation (3).^[^ ^49^ ^]^

(3) $$\mathrm{Wound}\;\mathrm{healing}\;\mathrm{ratio}\left(\%\right)=\left(A_{0}-A_{t}\right)/A_{0}\times 100\%$$

where *A*_0_ is the initial wound area and *A*_t_ represents the wound area at the desired time (*t*).

### ROS‐Scavenging Capability at the Cellular Level

Six groups (control, model, PMPB NCs [50 µg•mL^−1^], PMPB NC [100 µg•mL^−1^], Apr@PMPB NCs [50 µg•mL^−1^], Apr@PMPB NCs [100 µg•mL^−1^]) were set to explore the ROS‐scavenging capabilities at cellular levels using the DCFH‐DA probe. Raw 264.7 cells were seeded in confocal dishes at an appropriate concentration for 12 h. Except for the control group, cells in the other five groups were stimulated with LPS (15 ng•mL^−1^) for 12 h and later incubated with relevant solution for another 12 h. Subsequently, cells were rinsed with PBS and stained with a DCFH‐DA probe for 20 min. The in vivo ROS‐scavenging ability was observed using a confocal laser‐scanning microscope (CLSM) and the mean fluorescence intensity was analyzed by ImageJ software.

### In Vivo Therapeutic Efficiency and Biosafety Analyses

Healthy Kunming mice (≈20 g) were purchased and raised at Laboratory Animal Center, Shanghai Tenth People's Hospital. All animal experiments were in accordance with the guidelines of the Regional Ethics Committee for Animal Experiments. To establish the chemotherapy‐rooted OM model, doxorubicin (DOX) at a dose of 5 mg•kg^−1^ body weight was administered by tail vein injection for the mice on days 1, 3, and 5 of the experiment to duplicate the immunosuppression microenvironment of chemotherapy. On days 6 and 7, mice were anesthetized, and then their buccal mucosa was exposed to a glass rod that was pre‐immersed in 36% acetic acid for 1 min so as to obtain the ultimate OM model on mouse. After the successful establishment of the OM model on Kunming mice, PMPB@S and Apr@PMPB@S buccal tablets were applied to treat OM, and the OM‐Kunming mice undergoing no treatment were set as the model group (*n* = 5). The control group was healthy Kunming mice without OM. The buccal tablets were applied successively for 3 days in total.

After the experiment, the animals were sacrificed and the buccal mucosa around OM was collected for histological and immunohistochemical analysis. Immunofluorescence staining of CK 5 and CK 13 was performed to evaluate the regeneration of epithelium cells, where CK 5 mainly stained the surface layer, and CK 13 targeted the intermediate layer. The level of macrophage polarization was assessed by immunofluorescence staining (CD 206 and CD 86). The major organs (the heart, liver, spleen, lung, and kidney) of the animals were also harvested, embedded in paraffin, sectioned, and stained with H&E for histological analysis.

### Statistical Analysis

Data were presented as the means ± SD. One‐way analysis of variance (ANOVA) was used for comparisons among multiple groups. The differences were considered statistically significant when the *p*‐values were < 0.05 (*p < 0.05, **p < 0.01, ***p < 0.001). GraphPad Prism 9.5 was used for statistical analysis.

### Ethics Approval and Consent to Participate

All animal experiments were performed according to protocols approved by the Laboratory Animal Center of Shanghai Tenth People's Hospital and were in accordance with the policies of the National Ministry of Health (Approval number: SHDSYY‐2021‐3429‐2131158).

## Conflict of Interest

The authors declare no conflict of interest.

## Author Contributions

Y.Z. and T.W. contributed equally to this work. K.Z. designed the project; K.Z. and T.W. conceived and proposed the novelty and paper structure. Y.Z., T.W., F.C., X.D., C.Z., Q.P., C.L., and Y.Z. performed the experiments; Y.Z., T.W., F.C., and K.Z. analyzed the data. Y.Z. and T.W. wrote this manuscript; K.Z. revised and organized this manuscript. K.Z., F.C., and C.L. supported this project. K.Z. supervised the project and all authors commented on this manuscript.

## Supporting information

Supporting Information

## Data Availability

The data that support the findings of this study are available from the corresponding author upon reasonable request.

## References

[1] K. K.‐F. Cheng , J. Clin. Nurs. 2007, 16, 2114. 17313536 10.1111/j.1365-2702.2006.01618.x

[2] H. Y. Sroussi , J. B. Epstein , R.‐J. Bensadoun , D. P. Saunders , R. V. Lalla , C. A. Migliorati , N. Heaivilin , Z. S. Zumsteg , Cancer Med. 2017, 6, 2918. 29071801 10.1002/cam4.1221 PMC5727249

[3] D. De Ruysscher , G. Niedermann , N. G. Burnet , S. Siva , A. W. M. Lee , F. Hegi‐Johnson , Nat. Rev. Dis. Primers 2019, 5, 13. 30792503 10.1038/s41572-019-0064-5

[4] Y.‐Q. Liu , X.‐L. Wang , D.‐H. He , Y.‐X. Cheng , Phytomedicine 2021, 80, 153402. 33203590 10.1016/j.phymed.2020.153402

[5] P. Asikainen , T. J. Ruotsalainen , J. J. W. Mikkonen , A. Koistinen , C. Ten Bruggenkate , A. M. Kullaa , Med. Hypotheses 2012, 78, 790. 22465465 10.1016/j.mehy.2012.03.009

[6] J.‐L. Pico , A. Avila‐Garavito , P. Naccache , Oncologist 1998, 3, 446. 10388137

[7] R. V. Lalla , S. T. Sonis , D. E. Peterson , Dent. Clin. North Am. 2008, 52, 61. 18154865 10.1016/j.cden.2007.10.002 PMC2266835

[8] P. Riley , A. M. Glenny , H. V. Worthington , A. Littlewood , J. E. Clarkson , M. G. McCabe , Cochrane Database Syst. Rev. 2015, 2015, CD011552. 26695736 10.1002/14651858.CD011552.pub2 PMC8915172

[9] S. Elad , N. Yarom , Y. Zadik , M. Kuten‐Shorrer , S. T. Sonis , Ca–Cancer J. Clin. 2022, 72, 57. 34714553 10.3322/caac.21704

[10] G. Hatemi , A. Mahr , Y. Ishigatsubo , Y.‐W. Song , M. Takeno , D. Kim , M. Melikoglu , S. Cheng , S. Mccue , M. Paris , M. Chen , Y. Yazici , N. Engl. J. Med. 2019, 381, 1918. 31722152 10.1056/NEJMoa1816594

[11] J. Ouyang , Z. Zhang , B. Deng , J. Liu , L. Wang , H. Liu , S. Koo , S. Chen , Y. Li , A. V. Yaremenko , X. Huang , W. Chen , Y. Lee , W. Tao , Mater. Today 2023, 62, 296.

[12] J. L. Murdock , D. J. Reeves , J. Oncol. Pharm. Pract. 2020, 26, 521. 31142234 10.1177/1078155219850298

[13] A. R‐Caballeroa , D. T‐Lagaresa , M. R‐Garcı´aa , J. P‐Iba´n˜ezb , D. G‐Padillac , J. L. Gz‐Pe´reza , Int. J. Infect. Dis. 2012, 41, 225.

[14] P. Makvandi , U. Josic , M. Delfi , F. Pinelli , V. Jahed , E. Kaya , M. Ashrafizadeh , A. Zarepour , F. Rossi , A. Zarrabi , T. Agarwal , E. N. Zare , M. Ghomi , T. Kumar Maiti , L. Breschi , F. R. Tay , Adv. Sci. 2021, 8, 2004014. 10.1002/advs.202004014 PMC8061367 33898183

[15] J. Zhou , C. Fang , C. Rong , T. Luo , J. Liu , K. Zhang , Smart Mater. Med. 2023, 4, 427.

[16] V. F. Patel , F. Liu , M. B. Brown , J. Controlled Release 2012, 161, 746. 10.1016/j.jconrel.2012.05.026 22626941

[17] V. F. Patel , F. Liu , M. B. Brown , J. Controlled Release 2011, 153, 106. 10.1016/j.jconrel.2011.01.027 21300115

[18] S. Hu , X. Pei , L. Duan , Z. Zhu , Y. Liu , J. Chen , T. Chen , P. Ji , Q. Wan , J. Wang , Nat. Commun. 2021, 12, 1689. 33727548 10.1038/s41467-021-21989-5 PMC7966365

[19] J. Xu , S. Strandman , J. X. X. Zhu , J. Barralet , M. Cerruti , Biomaterials 2015, 37, 395. 25453967 10.1016/j.biomaterials.2014.10.024

[20] J. Xing , Y. Ding , X. Zheng , P. Yu , M. Qin , R. Qiu , Y. Li , S. Shang , J. Xie , J. Li , Chem. Eng. J. 2022, 444, 136580.

[21] Y. Cheng , S. K. Qin , Y. P. Chen , L. H. Dong , X. D. Sun , S. Y. Yu , S. K. Wu , OncoTargets Ther. 2018, 11, 8555. 10.2147/OTT.S185915 PMC6280986 30584316

[22] M. Chen , H. Liao , Z. Bu , D. Wang , C. Fang , X. Liang , H. Li , J. Liu , K. Zhang , D. Su , Chem. Eng. J. 2022, 441, 136030.

[23] Q. Zhang , L. Song , K. Zhang , Mater. Chem. Front. 2023, 7, 44.

[24] J. Zhong , X. Yang , S. Gao , J. Luo , J. Xiang , G. Li , Y. Liang , L. Tang , C. Qian , J. Zhou , L. Zheng , K. Zhang , J. Zhao , Adv. Funct. Mater. 2022, 33, 2209399.

[25] Z. Shao , T. Yin , J. Jiang , Y. He , T. Xiang , S. Zhou , Bioact. Mater. 2023, 20, 561. 35846841 10.1016/j.bioactmat.2022.06.018 PMC9254353

[26] W. Zhang , S. Hu , J.‐J. Yin , W. He , W. Lu , M. Ma , N. Gu , Y. Zhang , J. Am. Chem. Soc. 2016, 138, 5860. 26918394 10.1021/jacs.5b12070

[27] M. A. Komkova , E. E. Karyakina , A. A. Karyakin , J. Am. Chem. Soc. 2018, 140, 11302. 30118222 10.1021/jacs.8b05223

[28] K. Zhang , M. Tu , W. Gao , X. Cai , F. Song , Z. Chen , Q. Zhang , J. Wang , C. Jin , J. Shi , X. Yang , Y. Zhu , W. Gu , B. Hu , Y. Zheng , H. Zhang , M. Tian , Nano Lett. 2019, 19, 2812. 30908916 10.1021/acs.nanolett.8b04729

[29] Q. Hu , P. S. Katti , Z. Gu , Nanoscale 2014, 6, 12273. 25251024 10.1039/c4nr04249b PMC4425417

[30] J. Mu , J. Lin , P. Huang , X. Chen , Chem. Soc. Rev. 2018, 47, 5554. 29856446 10.1039/c7cs00663b PMC6066418

[31] Y. Zhang , Y. Yin , W. Zhang , H. Li , T. Wang , H. Yin , L. Sun , C. Su , K. Zhang , H. Xu , J. Nanobiotechnol. 2021, 19, 161. 10.1186/s12951-021-00897-2 PMC8166117 34059078

[32] Y. Yin , X. Jiang , L. Sun , H. Li , C. Su , Y. Zhang , G. Xu , X. Li , C. Zhao , Y. Chen , H. Xu , K. Zhang , Nano Today 2021, 36, 101009.

[33] W. Zhang , J. Wang , Z. Xie , H. Zou , Q. Chen , L. Xu , L. Hu , N. Fang , J. Xu , J. Zhou , J. Liu , H. Ran , Z. Wang , Y. Zhang , D. Guo , Small 2022, 18, 2106252. 10.1002/smll.202106252 35246943

[34] M. Lian , Z. Xue , X. Qiao , C. Liu , S. Zhang , X. Li , C. Huang , Q. Song , W. Yang , X. Chen , T. Wang , Chem 2019, 5, 2378.

[35] T. Liu , B. Xiao , F. Xiang , J. Tan , Z. Chen , X. Zhang , C. Wu , Z. Mao , G. Luo , X. Chen , J. Deng , Nat. Commun. 2020, 11, 2788. 32493916 10.1038/s41467-020-16544-7 PMC7270130

[36] J. Zhou , W. Liu , X. Zhao , Y. Xian , W. Wu , X. Zhang , N. Zhao , F.‐J. Xu , C. Wang , Adv. Sci. 2021, 8, 2100505. 10.1002/advs.202100505 PMC8529445 34414693

[37] M. Liu , L. Huang , X. Xu , X. Wei , X. Yang , X. Li , B. Wang , Y. Xu , L. Li , Z. Yang , ACS Nano 2022, 16, 9479. 35713471 10.1021/acsnano.2c02518

[38] X. Pan , N. Wu , S. Tian , J. Guo , C. Wang , Y. Sun , Z. Huang , F. Chen , Q. Wu , Y. Jing , Z. Yin , B. Zhao , X. Xiong , H. Liu , D. Zhou , Adv. Funct. Mater. 2022, 32, 2112145.

[39] F. Dong , Y. Chu , M. He , Y. Zhang , W. Li , P. K. Liaw , B. Wang , L. Luo , Y. Su , R. O. Ritchie , X. Yuan , J. Mater. Sci. Technol. 2022, 102, 36.

[40] J. P. Wu , Y. Lin , F. H. Duan , Q. Chen , H. T. Wang , N. Li , J. L. Wen , J. Pan , L. Liu , J. Mater. Sci. Technol. 2023, 163, 140.

[41] J. H. Ryu , J. S. Choi , E. Park , M. R. Eom , S. Jo , M. S. Lee , S. K. Kwon , H. Lee , J. Controlled Release 2020, 317, 57. 10.1016/j.jconrel.2019.11.006 31712088

[42] K. Wu , M. Fu , Y. Zhao , E. Gerhard , Y. Li , J. Yang , J. Guo , Bioact. Mater. 2023, 20, 93. 35633874 10.1016/j.bioactmat.2022.05.017 PMC9131258

[43] H. An , Z. Gu , L. Zhou , S. Liu , C. Li , M. Zhang , Y. Xu , P. Zhang , Y. Wen , Acta Biomater. 2022, 149, 126. 35840105 10.1016/j.actbio.2022.07.016

[44] W. Zhang , B. Bao , F. Jiang , Y. Zhang , R. Zhou , Y. Lu , S. Lin , Q. Lin , X. Jiang , L. Zhu , Adv. Mater. 2021, 33, 2105667. 10.1002/adma.202105667 34605063

[45] M. Wang , X. Huang , H. Zheng , Y. Tang , K. Zeng , L. Shao , L. Li , J. Controlled Release 2021, 337, 236. 10.1016/j.jconrel.2021.07.017 34273419

[46] D. S. W. Benoit , K. R. Sims , D. Fraser , ACS Nano 2019, 13, 4869. 31033283 10.1021/acsnano.9b02816 PMC6707515

[47] L. Lu , T. Wang , C. Fang , L. Song , C. Qian , Z. Lv , Y. Fang , X. Liu , X. Yu , X. Xu , C. Su , F. Chen , K. Zhang , ACS Appl. Mater. Interfaces 2022, 14, 36462. 35939287 10.1021/acsami.2c09443

[48] Q. Zeng , Y. Qian , Y. Huang , F. Ding , X. Qi , J. Shen , Bioact. Mater. 2021, 6, 2647. 33665497 10.1016/j.bioactmat.2021.01.035 PMC7890098

[49] W. Liu , M. Wang , W. Cheng , W. Niu , M. Chen , M. Luo , C. Xie , T. Leng , L. Zhang , B. Lei , Bioact. Mater. 2021, 6, 721. 33005834 10.1016/j.bioactmat.2020.09.008 PMC7516176

