# Supplementary material for: Salivary Amylase‐Responsive Buccal Tablets Wipe Out Chemotherapy‐Rooted Refractory Oral Mucositis
Source: Adv Sci (Weinh). 2024 Jan 16;11(11):2308439. doi: 10.1002/advs.202308439 (PMC10962474; doi:10.1002/advs.202308439)
Supplement: Supplementary file 1 — Supporting Information [file ADVS-11-2308439-s001.pdf]

ADVANCED SCIENCE
Open Access

Supporting Information

for *Adv. Sci.*, DOI 10.1002/advs.202308439

Salivary Amylase-Responsive Buccal Tablets Wipe Out Chemotherapy-Rooted Refractory Oral Mucositis

Yan Zhang, Taixia Wang, Xiulin Dong, Chunyan Zhu, Qiuxia Peng, Chang Liu, Yifeng Zhang, Fubo Chen* and Kun Zhang**

Supporting Information for

Salivary Amylase-Responsive Buccal Tablets Wipe Out Chemotherapy-Rooted Refractory Oral Mucositis

Yan Zhang,[#] Taixia Wang,[#] Xiulin Dong, Chunyan Zhu, Qiuxia Peng, Chang Liu, Yifeng Zhang, Fubo Chen* and Kun Zhang**

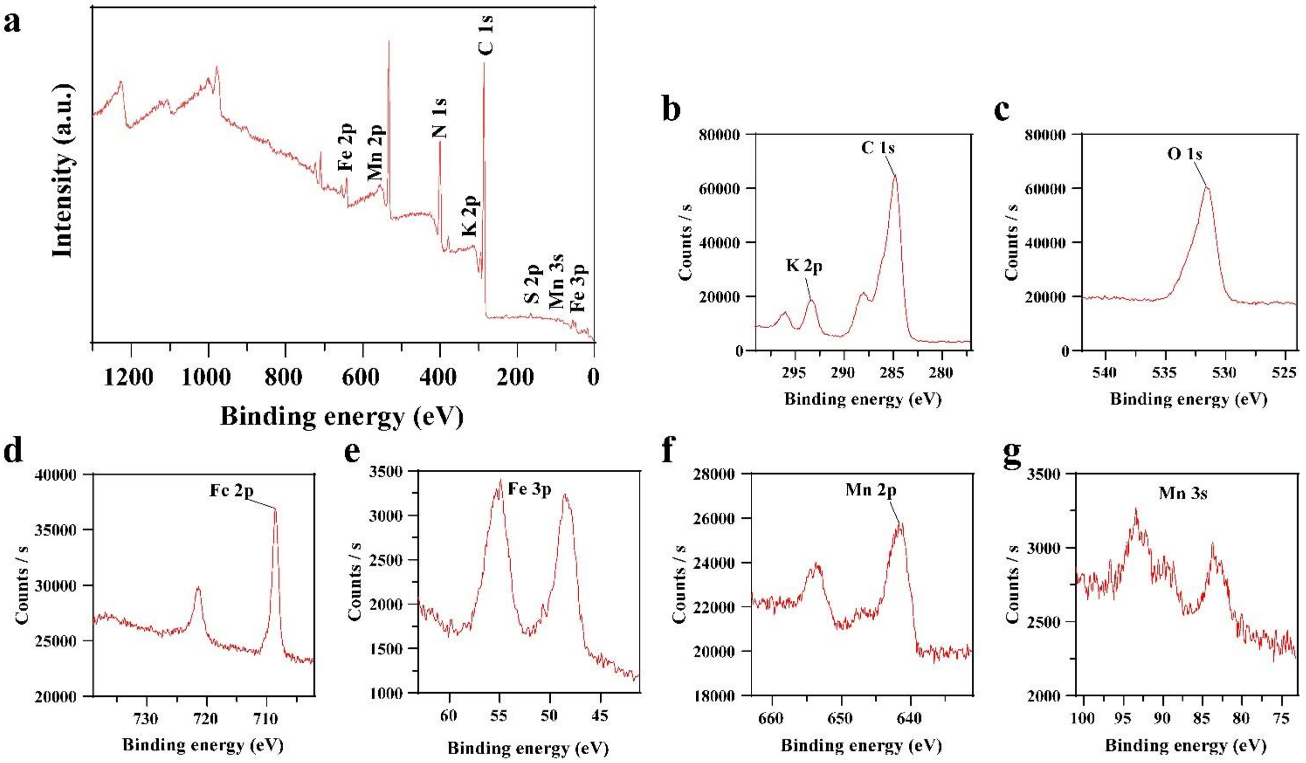

Figure S1. (a) XPS spectral of PMPB NCs. (b) Narrow-window XPS spectra of C. (c) Narrow-window XPS spectra of O. (d-g) Narrow-window XPS spectra of Fe and Mn atoms in PMPB NCs at different electron orbits: (d) Fe 2p, (e) Fe3p, (f) Mn 2p and (g) Mn 3s.

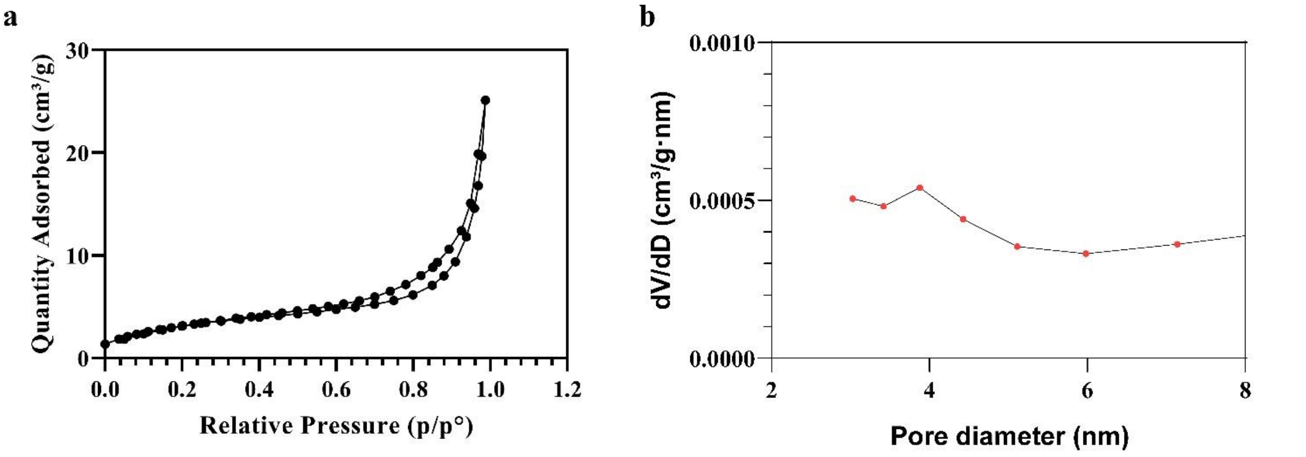

Figure S2. (a) N_2 adsorption and desorption isotherms of PMPB NCs. (b) Pore diameter distributions of PMPB NCs.

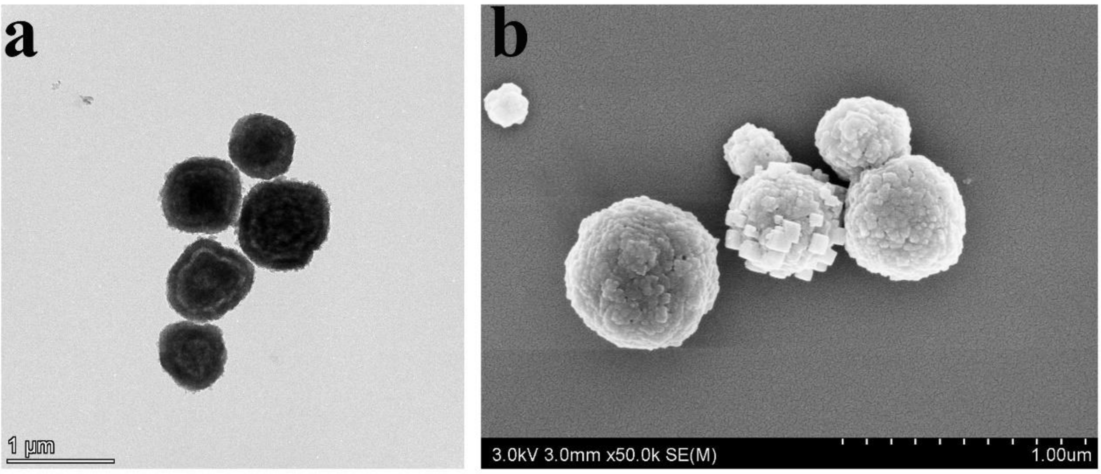

Figure S3. (a) TEM and (b) SEM images of Apr@PMPB NCs.

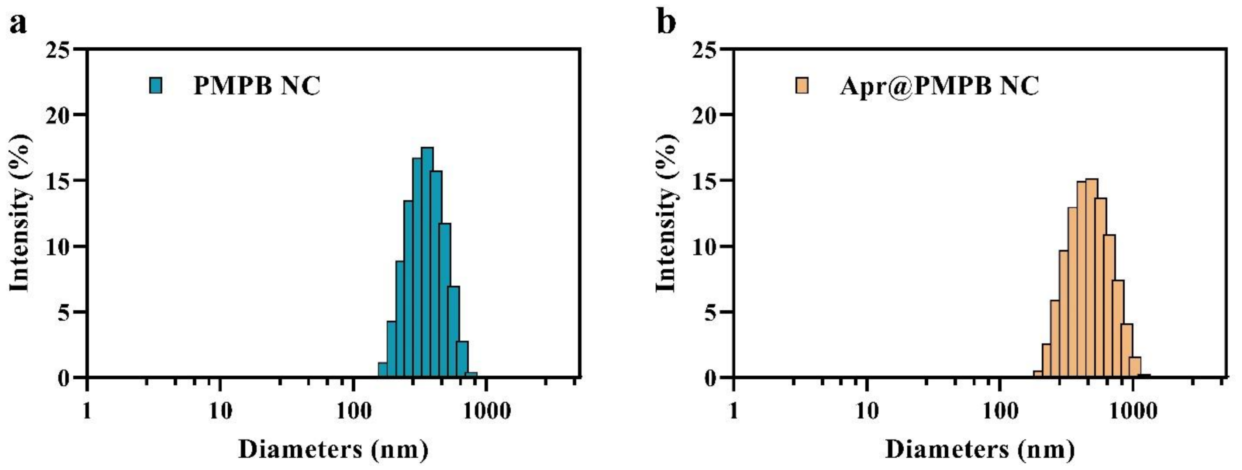

Figure S4. (a) Hydrodynamic diameter distribution of PMPB NCs. (b) Hydrodynamic diameter distribution of Apr@PMPB NCs.

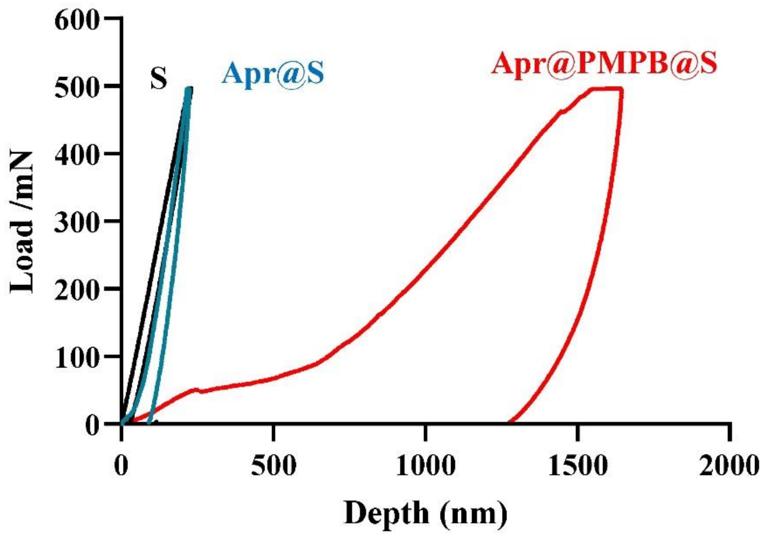

Figure S5. Nanoindentation load-displacement (P-h) curves of S, Apr@S, Apr@PMPB@S.

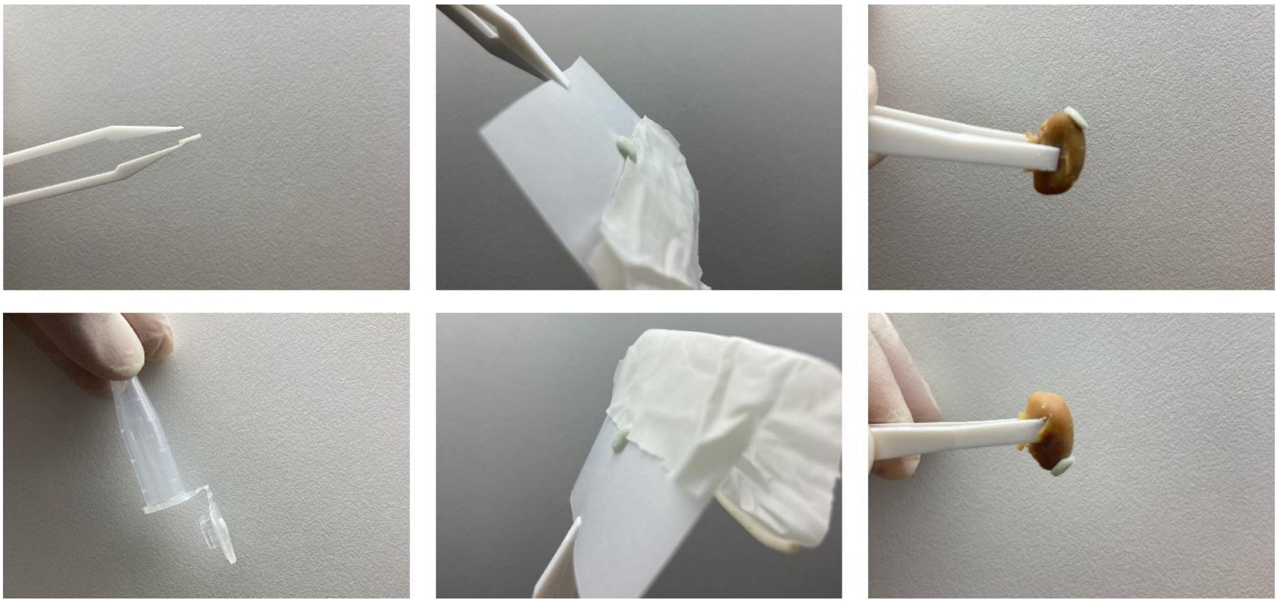

Figure S6. Digital photos of Apr@PMPB@S buccal tablets onto different surfaces.

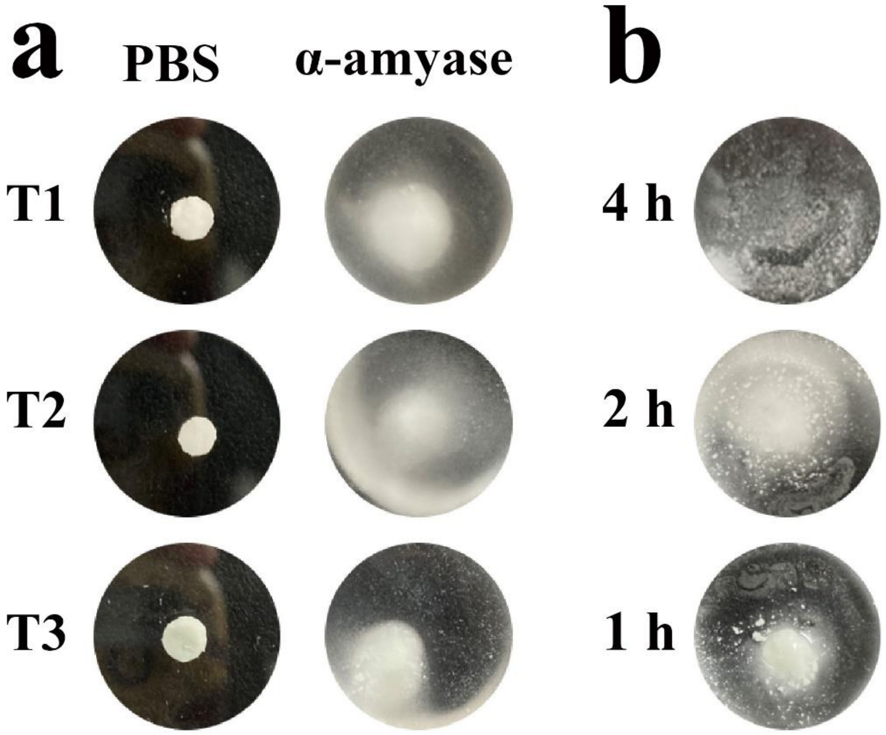

Figure S7. (a) Photographs of Apr@PMPB@S tablets in PBS and α-amyase for 1 h, respectively; (b) Time-dependent photographs of Apr@PMPB@S tablets in α-amyase.

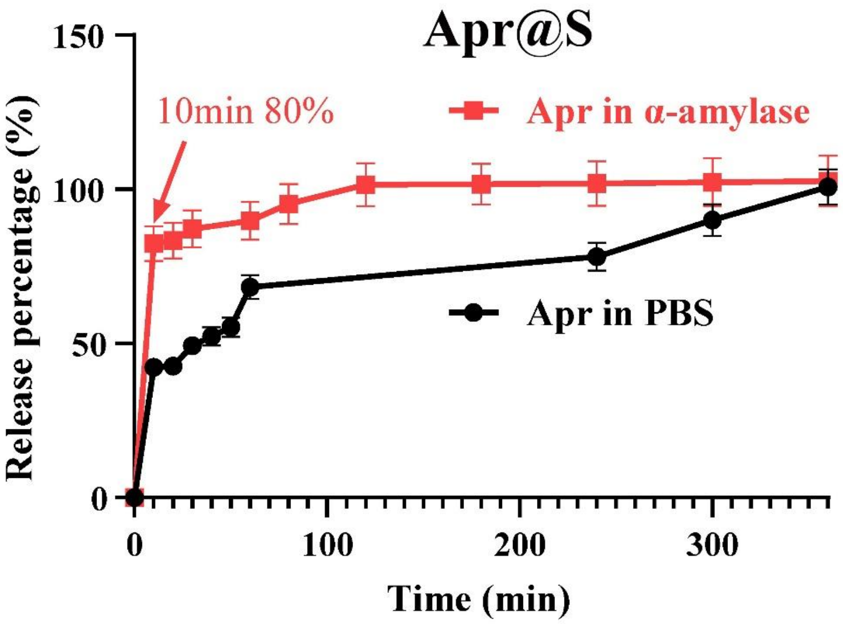

Figure S8. Time-dependent release profiles of Apr from Apr@S buccal tablets with and without adding α-amylase. Data are expressed as mean±SD (n=3).

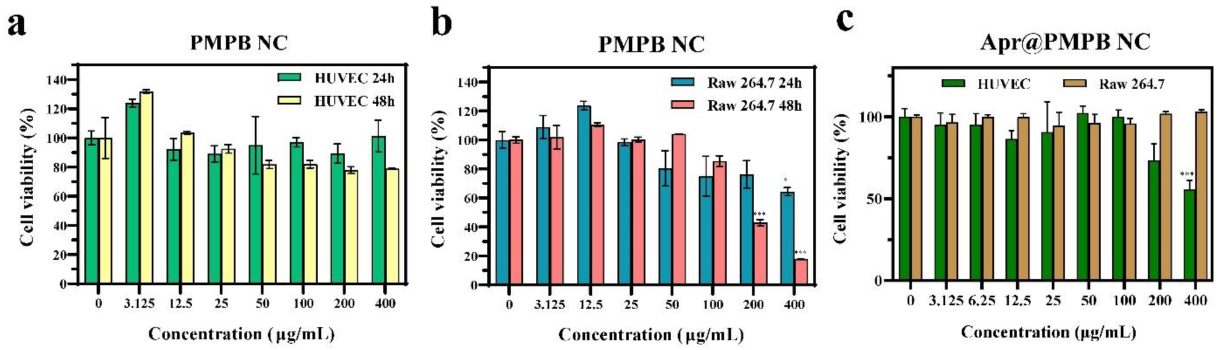

Figure S9. (a) Cell viability of HUVECs treated with PMPB NC for 24 and 48 h. (b) Cell viability of Raw 264.7 cells treated with PMPB NC for 24 h and 48 h. (c) Cell viability of HUVEC and Raw 264.7 cells treated with Apr@PMPB NC for 24 h. Data are expressed as mean±SD (n=5). One-way analysis of variance (ANOVA) was used for comparisons among multiple groups (*p < 0.05, **p < 0.01, ***p < 0.001).

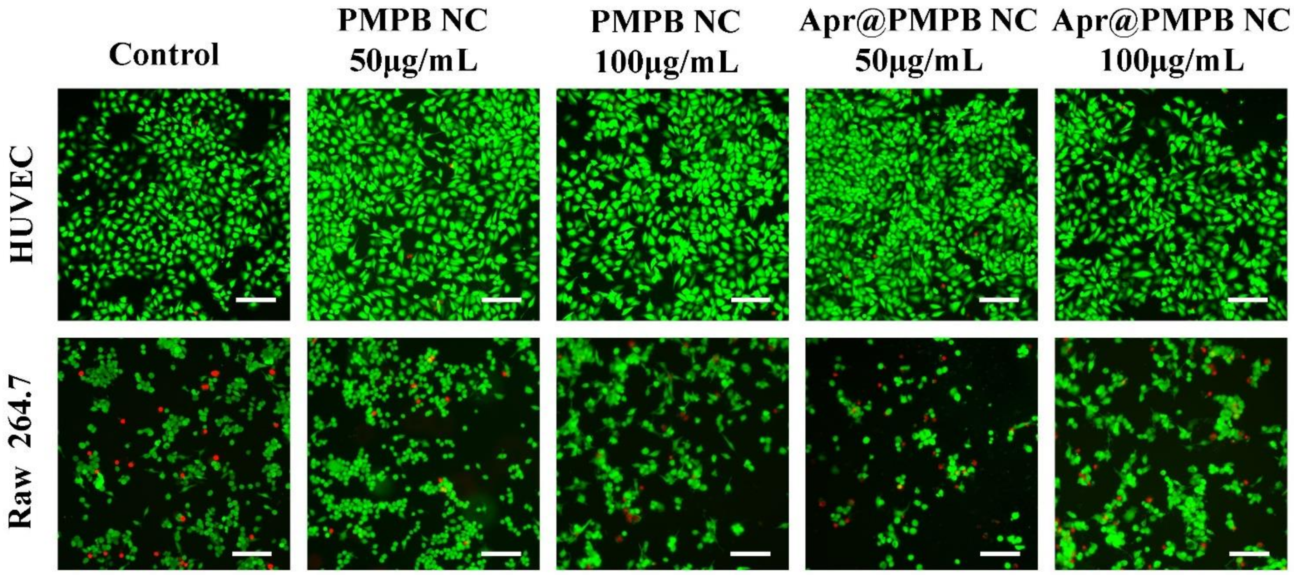

Figure S10. CLSM images of HUVECs and Raw 264.7 cells stained with Live/Dead after treatments with PMPB NC and Apr@PMPB NC with varied concentrations (50 μg•mL^{-1}, 100 μg•mL^{-1}), scale bar: 200 μm.

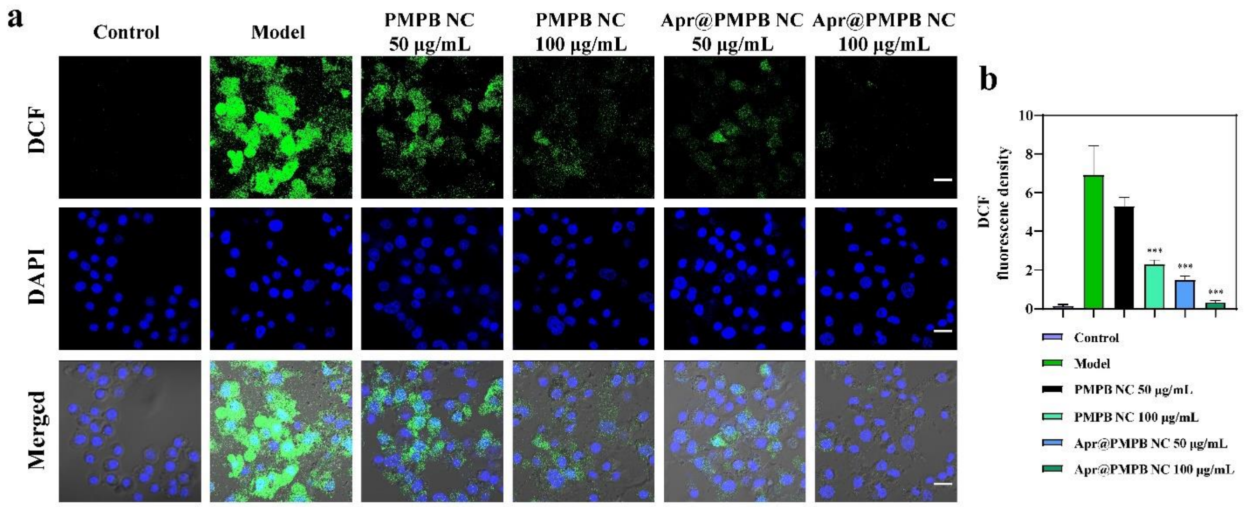

Figure S11. CLSM images (a) and quantitative data (b) of Raw 264.7 cells stained with the ROS indicator, i.e., DCFH-DA probe after different treatment, and scale bar: 20 μm. Data are expressed as mean±SD (n=3). ANOVA test was used for comparisons among multiple groups (*p < 0.05, **p < 0.01, ***p < 0.001).

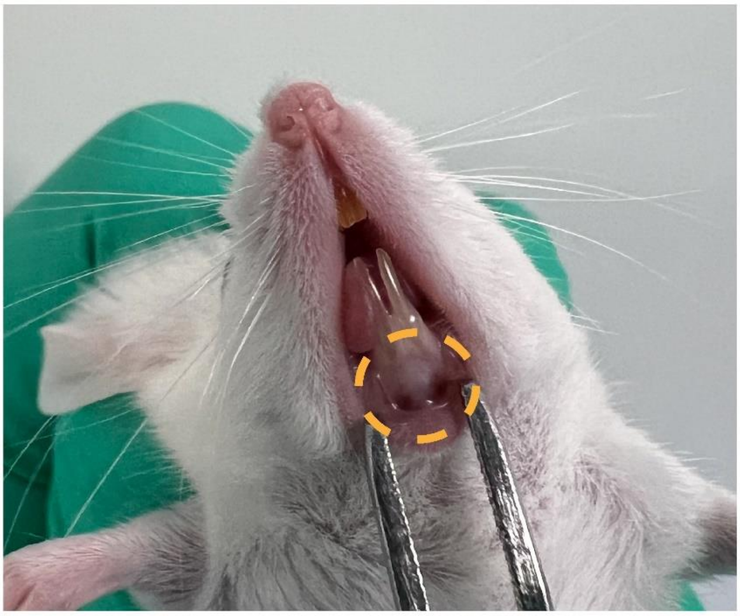

Figure S12. OM model established on Kunming mice.

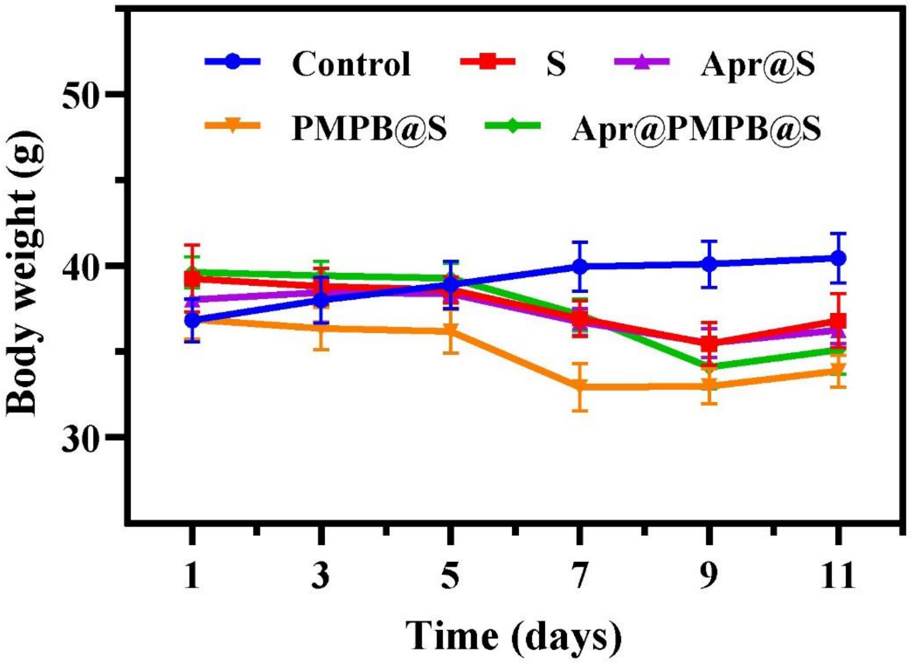

Figure S13. Time-dependent body weight curves of Kunming mice that experienced different treatments. Data were expressed as mean value ± SD (n=5).

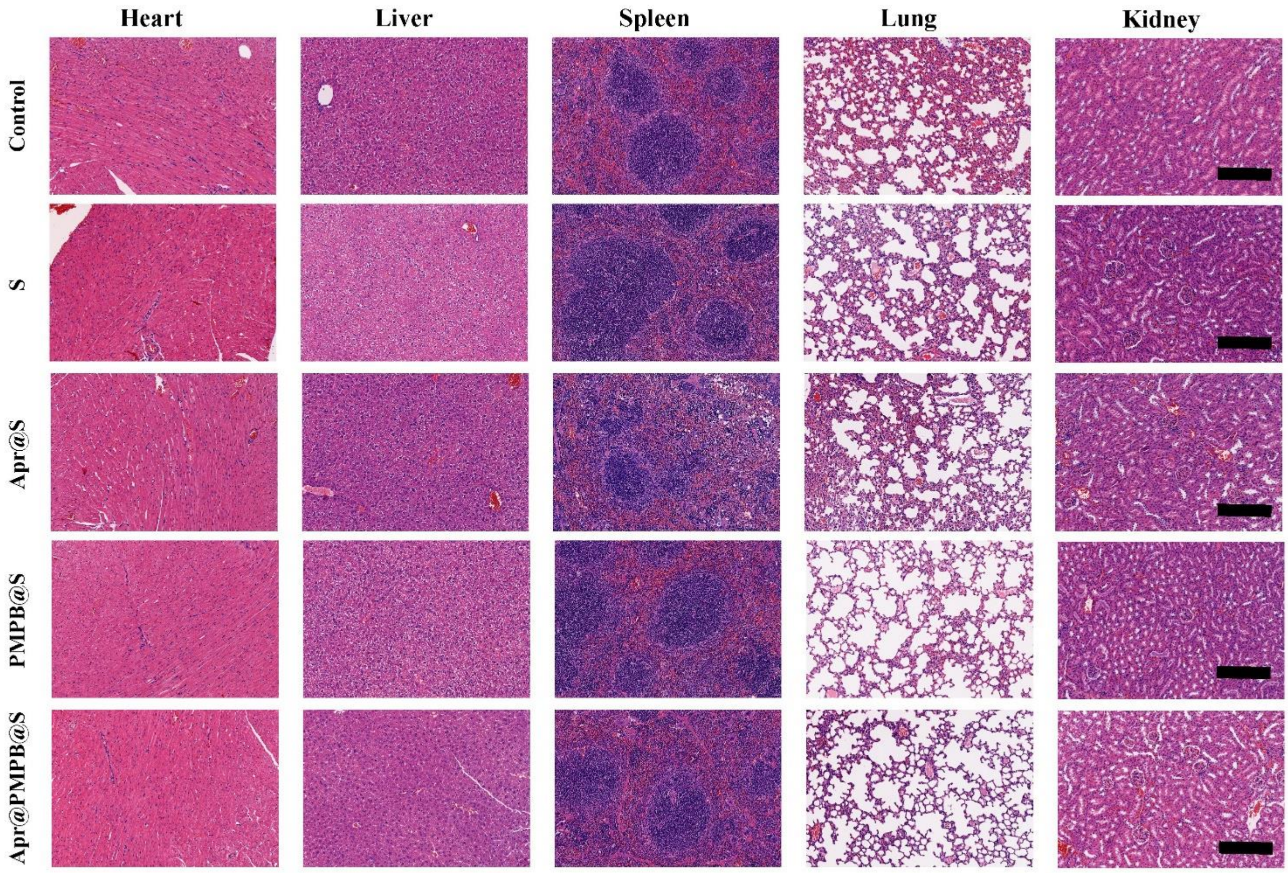

Figure S14. H&E staining images of major organs (the heart, liver, spleen, lung, and kidney) after different treatments, scale bar: 200 μm. Data are expressed as mean±SD (n=5).

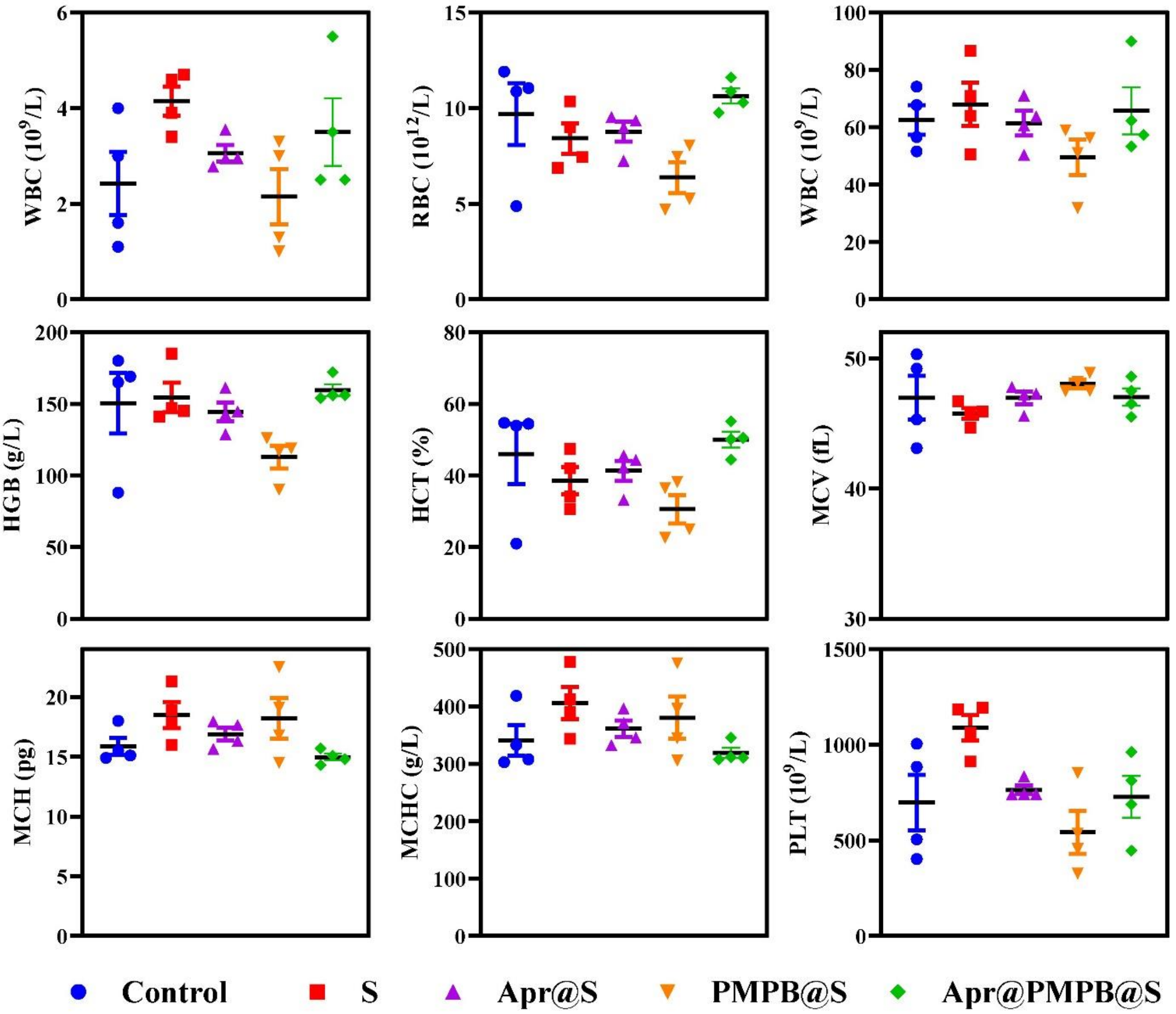

Figure S15. Blood biochemical indexes of mice in four groups. Data were expressed as mean value ± SD (n=4).
